# Assessing knowledge levels of intensive care unit nurses and doctors regarding drug administration via enteral feeding tubes: a survey study

**DOI:** 10.55730/1300-0144.5957

**Published:** 2025-02-02

**Authors:** Ayşe Gül KOÇOĞLU KINAL, Yunus Emre AYHAN, Aslınur ALBAYRAK

**Affiliations:** 1 Intensive Care Unit, Dr. Siyami Ersek Thoracic and Cardiovascular Surgery Training and Research Hospital, İstanbul, Turkiye; 2 Department of Clinical Pharmacy, Prof. Dr. Cemil Taşcıoğlu City Hospital, İstanbul, Turkiye; 3 Department of Clinical Pharmacy, Süleyman Demirel University, Faculty of Pharmacy, Isparta, Turkiye

**Keywords:** Enteral feeding tubes, intensive care unit, dosage form, doctor, nurse

## Abstract

**Background/aim:**

Knowledge deficiency regarding appropriate drug administration through enteral feeding tubes (EFTs) is common in intensive care units (ICUs). The aim of this study is to evaluate the knowledge levels of nurses and doctors in ICUs about drug administration via EFTs.

**Materials and methods:**

This study was conducted as a cross-sectional online survey. Survey questions were created using Google Forms and distributed to nurses and doctors in various ICUs across hospitals in İstanbul, Türkiye. The researchers designed the survey questions based on literature reviews and existing examples. The survey consisted of three sections and a total of 25 questions: the first section included seven demographic questions, while the second and third sections focused on participants’ knowledge regarding drug administration via EFT and the selection of appropriate dosage forms, respectively.

**Results:**

The online survey form was sent to 400 healthcare workers in ICUs, and 221 (55.2%) completed the survey. Among the participants, 66 (29.9%) were male, and 112 (50.6%) were doctors. There was no significant difference in the mean (± SD) of correct answers to 9 questions on drug administration between doctors (5.4 ± 1.3) and nurses (5.3 ± 1.4) (p = 0.471). In the mean (± SD) of correct answers to 9 questions on dosage form selection, doctors (3.9 ± 2.1) had higher scores than nurses (2.7 ± 1.9) (p < 0.001). The mean (± SD) of the responses given to all questions was found to be higher in doctors (9.4 ± 2.9) than in nurses (8 ± 2.7) (p < 0.001).

**Conclusion:**

In ICUs, the knowledge of nurses and doctors about drug administration and dosage form selection via EFT is at a low to moderate level. Enhancing collaboration among healthcare professionals may be effective in bridging this knowledge gap.

## 1. Introduction

In intensive care units (ICUs), oral feeding of patients is not possible due to intubation, deep sedation, and multiple comorbidities [1,2]. In critically ill patients, nutrition and drug administration, which are important parts of medical treatment, are usually performed parenterally or enterally [3,4].

In clinics, enteral feeding tubes (EFT) are widely used for drug administration [5]. Therefore, it is essential to evaluate the appropriateness of drug administration via EFT in critically ill patients where multiple drug use is frequently seen [6]. Inappropriate dosage form selection for administration via EFT, incorrect administration technique, physicochemical incompatibility, drug-nutrient interaction, and tube occlusion are frequently encountered problems in drug administration via EFT [6–8]. Drug administration via EFT stands out as a frequently encountered problem in geriatric, neurological, and critically ill patients [9–13]. Previous studies have listed factors such as inadequate dosage form knowledge of healthcare professionals and lack of cooperation of healthcare professionals in this process as the leading causes of EFT-related medication administration errors (EFTRMAEs) [4,11,14–17].

Improving the quality of medication administration with EFT is very important for critically ill patients. When developing a nutritional support plan, it is essential to identify potential problems with the patient’s treatment and to evaluate the appropriateness of medications administered via EFT. Although guidelines exist to optimize medication administration with EFT, adherence to these guidelines is low [18]. The prevalence of EFTRMAEs poses a severe challenge to patient management, especially in ICU settings [10,13,14]. Previous studies have shown that EFTRMAEs are common in hospitalized patients, with a frequency ranging from 43% to 80% [5,12,13,18–22]. Walther et al., in a study conducted in neurology and neurosurgery ICUs on drug administrations and prescriptions via EFT, reported that nursing staff mainly reported inappropriate drug administrations via EFT (44%) related to modified release drugs [13]. Previous studies have observed that healthcare professionals participating in an education program did not have sufficient basic knowledge about drug administration rules via EFT [16,17,23,24]. Alhashemi et al. also reported in similar studies that <20% of participants had sufficient awareness about crushing solid dosage forms before the education program [24].

Although drug administration procedures are primarily nurses’ duty, many studies in the literature have emphasized that prescribing doctors is the primary responsibility for selecting the dosage form to reduce EFTRMAEs [23,25]. Being aware of this fact, this study aimed to question the knowledge levels of nurses and doctors in ICUs. This study aimed to evaluate the knowledge levels of nurses and doctors working in ICUs regarding drug administration via EFT and dosage form selection. Including ICU doctors in the study evaluating this level of knowledge will contribute to the literature.

## 2. Materials and methods

### 2.1. Study design and participants

This cross-sectional online survey study was conducted between October 15 and 22, 2024. It was conducted on nurses and doctors who are over 18 years of age and actively working in ICUs. The study was conducted in the ICUs of 5 different education and research hospitals in İstanbul/Türkiye. Survey questions were created with Google Forms and distributed online to ICU doctors and nurses via phone and e-mail.

The study received ethical approval from the Süleyman Demirel University Health Sciences Ethics Committee (Meeting-decision no: 80/7, Date: 27.09.2024). All procedures were conducted in accordance with the principles of the 1964 Helsinki Declaration and its later amendments. The online survey included a hyperlink to the consent form for participants’ personal use. Consent forms were cross-referenced to verify participant identities.

### 2.2. Data collection and survey questions

The researchers created the survey questions by examining the Clinical Enteral Parenteral Nutrition Association (KEPAN) Enteral Nutrition Guide and a comprehensive and in-depth literature search specific to the objectives of the current study to assess the level of knowledge regarding the appropriateness of the application methods and dosage forms of drugs administered via EFT [19,23,24,26,27]. Clinical pharmacists and intensive care specialists evaluated the structured items of the survey for restructuring, relevance, and accuracy and went through a translation process from English to Turkish and vice versa.

To determine the survey’s reliability, validity, and clarity, a pilot test-retest study was conducted on 30 intensive care nurses and doctors from an education and research hospital, which were not included in the main study. As internal and external consistency measures for the questions, Cronbach’s alpha coefficient was calculated as 0.65 and kappa coefficient as 0.6, respectively. The questions were generally found to be understandable.

The link to the survey questions was distributed online via Google Forms as a phone message and e-mail. A consent form signed by each participant was approved electronically. The online survey included a hyperlink to the consent form for the participant’s personal use. The consent forms were cross-referenced to verify participant identities, and the survey form was designed to allow only one completion. The survey questions consisted of three sections and 25 questions in total.

1. The first section of the survey consisted of 7 questions, including demographic information such as age, sex, and professional experience, and questions aimed at assessing the participants’ professional and educational backgrounds.
2. The nine questions in the second part of the survey aim to evaluate the participants’ knowledge level about drug administration methods via EFTs. Participants are asked how more than one drug should be administered simultaneously, whether liquid dosage forms should be diluted, and whether drugs can be added to the enteral feeding formula. In addition, essential points such as the duration of feeding breaks before and after drug administration and washing the tube before and after drug administration are also evaluated.
3. The nine questions in the second part of the survey are designed to evaluate the knowledge level of nurses and doctors in dosage form selection. Participants are asked which dosage forms are appropriate for administration via EFTs. In this context, examples of extended-release, zero-order kinetic (ZOK), enteric-coated, and controlled-release tablets were given, and participants were questioned about their knowledge of dosage forms.

### 2.3. Assessment of knowledge level

A precise and objective measurement of the knowledge and performance of the healthcare team was provided by giving 1 point for correct answers and 0 points for incorrect answers. Accordingly, the participants in the survey were evaluated with a minimum score of 0 and a maximum score of 18. The correct answers given to the questions in the survey were presented as percentages and as the average scores for each area of the survey. The average correct answers to the 18 knowledge level questions were classified as low for 0–6 correct answers, medium for 7–12 correct answers, and high for 13–18 correct answers. The average correct answers for the two sections of 9 questions were classified as low for 0–3 correct answers, medium for 4–6 correct answers, and high for 7–9 correct answers. The correct answers of 0%–35% of the participants to all questions were classified as low, 36%–70% were classified as medium, and 71%–100% were classified as high.

### 2.4. Inclusion and exclusion criteria

The study included nurses and doctors who were 18 years of age or older and actively working in the ICU. Participants who answered the survey questions incompletely were excluded from the study.

### 2.5. Sample size

Since the number of nurses and doctors working in ICUs in İstanbul/Türkiye is unknown, surveys were distributed to 400 ICU nurses and doctors from 5 different hospitals. The minimum sample size was calculated as 197 with a 95% confidence interval and 5% error using the Raosoft sample size calculator. 1

### 2.6. Statistical analysis

The descriptive statistics, including the means, medians, standard deviations, interquartile ranges (IQR), counts, and percentages, were used to assess the central tendency and variability of the continuous variables. For categorical variables, frequencies and percentages are given. The Kolmogorov-Smirnov test was used to determine whether continuous variables followed a normal distribution. The result was nonparametric. The Mann-Whitney U test was used to compare continuous variables between two groups. Categorical data were compared via chi-square tests. A 95% confidence interval (CI) with a p-value less than 0.05 was considered statistically significant. Homogeneity or internal consistency of the knowledge questions was assessed with Cronbach’s alpha and kappa coefficients. The dataset was analyzed overall with the help of IBM SPSS Statistics for Windows, Version 29.0 (Armonk, New York: IBM Corp.).

## 3. Results

In the study, 400 healthcare professionals were invited to participate in the online survey, and 221 (55.2%) completed it. A total of 221 healthcare professionals were included in the study, 112 (50.6%) of whom were doctors and 109 (49.4%) were nurses. 66 (29.9%) of the participants were male, and the median age (IQR) was 35 (29–43) years. The median (IQR) years of professional experience of the doctors in the ICU was 7.5 (4–11) years, 5 (3–9) years more than the nurses (p = 0.006). The percentage of doctors who had never received education in the preparation and administration of medications using EFT (66.9%) was higher than the nurses (25.6%) (p < 0.001) (Table 1).

The percentages of participants who answered the survey questions correctly were evaluated. Accordingly, it was determined that doctors (61.6%) and nurses (58.7%) who answered the questions about drug administration correctly had a moderate level of knowledge (p = 0.471). Doctors (43.75%) who answered the questions about dosage form selection correctly had a moderate level of knowledge, and nurses (30.2%) had a low level of knowledge (p < 0.001). When all survey questions were evaluated, doctors (51.7%) and nurses (44.9%) had a moderate level of knowledge (p < 0.001) (Table 2). There was no significant difference between doctors (5.4 ± 1.3) and nurses (5.3 ± 1.4) in the mean (± SD) correct answers to 9 questions about drug administration from the survey questions (p = 0.471), and they were at a moderate level. The mean (±SD) of correct answers to 9 questions regarding dosage form selection from the survey questions was higher in doctors (3.9 ± 2.1) than in nurses (2.7 ± 1.9) and was at a low level (p < 0.001). The mean (±SD) of answers to all questions was higher in doctors (9.4 ± 2.9) than in nurses (8 ± 2.7) (p < 0.001) and was at a medium level (Table 3).

Factors affecting the number of correct answers of the participants were analyzed. Accordingly, specialist doctors were more likely to have a total score > 9 points than other healthcare professionals (odds ratio, 95% CI, p = 0.39 (0.22–0.68), 0.001). No statistically significant relationship was found between age, sex, total professional experience, ICU experience, and education status on drug administration with EFT, and a total correct answer score > 9 (p > 0.05).

## 4. Discussion

This study assessed the knowledge of nurses and doctors providing services in ICUs about drug administration and dosage forms via EFT. It is noteworthy that doctors have a higher level of knowledge about drug dosage form selection and all questions compared to nurses, and this situation emphasizes the importance of a multidisciplinary approach.

### 4.1. Knowledge levels of intensive care unit doctors and nurses about drug administration

Effective administration of drugs is vital to ensure optimal patient outcomes, especially in critical care settings such as ICU [2,28]. Drug administration via EFTs is the responsibility of nurses in many healthcare institutions [5,16,24,29], However, the majority of studies in the literature indicate that the knowledge level of nurses providing active service in ICUs about drug administration via EFT is inadequate [5,17,23,24,30]. In this study, doctors had a moderate level of knowledge in the section of the survey questions related to drug administration, similar to nurses, regarding correct response percentage and correct response average. However, there were no sufficient studies in the literature on the knowledge levels of ICU doctors regarding drug administration from EFT. To prevent EFT blockage and interaction risks, guidelines recommend washing the tube before and after drug administration and administering drugs separately [7,31]. Healthcare professionals demonstrated acceptable knowledge of preparing drugs separately, administering them individually, and flushing EFTs between administrations. However, doctors consistently outperformed nurses in these areas. However, the knowledge level of healthcare professionals in this study regarding the time to be left between drug administration and feeding was relatively low. Studies consistently highlight low adherence to flushing and separate administration guidelines, with only 5%–43% of practitioners flushing between medications and 18%–51% administering drugs separately [32,33,35,36]. On the other hand, a study by Mota et al. found that 30.7% of the nurses did not flush the tube between administrations when administering multiple medications, while the rate of nurses administering medications separately was 18.3% [24].

Medication administration guidelines recommend not adding medication directly to enteral feeding formulas [6]. Multiple studies, including the ASPEN survey, report low compliance with evidence-based guidelines, such as flushing tubes and administering medications separately [32,34]. In this study, it was seen that the participants had a moderate level of knowledge that drugs should not be added directly to the feeding formula. This is comparable to the literature. The literature also emphasizes that nurses should stop feeding patients 30 min before and after drug administration [6,7,37]. Sari et al. found that less than half of the nurses (44.8%) stopped enteral feeding at least 30 min before drug administration [32]. On the other hand, Guenter and Boullata found that this rate was 95% [34]. It seems that these inappropriate practices, which are widely stated in the literature, can be explained by the high rate of lack of knowledge.

Another critical issue regarding drug administration is the dilution of liquid drugs before administration via EFT. In previous studies, 43%–48% of the participants stated that they did not dilute liquid drugs before administration [32–34]. Similarly, in this study, almost half of the doctors and nurses stated that liquid drugs should be administered after dilution. However, the level of knowledge regarding another drug administration question, not to add liquid formulations directly to enteral feeding formula, is relatively low. This situation shows that healthcare professionals experience inconsistency in both knowledge and practice regarding the administration of liquid formulations via EFT. On the other hand, the fact that there is a very limited number of liquid formulations of drugs may be another reason explaining the lack of knowledge of the majority of nurses on this issue [23,367,^38^].

### 4.2. Knowledge levels of intensive care unit doctors and nurses regarding dosage forms

Doctors are responsible for drug selection during the drug prescription process. In addition, doctors should be careful because manufacturers and regulators have not evaluated oral drugs for use via EFT. This study asked healthcare professionals in ICUs about dosage forms, mainly controlled-release, extended-release, and capsules. Doctors demonstrated significantly higher knowledge of appropriate dosage forms than nurses, likely due to their prescribing authority. However, based on the findings of this study, it was observed that doctors did not have sufficient knowledge about dosage forms. In a survey conducted by Demirkan et al. in all units of a hospital, 14% of doctors and 2% of nurses stated that they ignored the appropriateness of drugs when they prescribed or administered drugs via EFT. 17% of nurses and 24% of doctors stated that they were aware that enteric-coated tablets should not be crushed. In addition, no statistically significant difference between doctors and nurses was found between a conventional tablet, a modified-release tablet, and an enteric-coated tablet in the survey [16]. However, in this current study conducted on ICU nurses and doctors, the knowledge levels of doctors about dosage forms were statistically significantly higher than that of the nurses. This situation can be explained by doctors being more careful about the dosage forms prescribed for critically ill patients in the ICU than doctors in other wards.

In the studies evaluating the dosage forms administered via EFT in the literature, it is seen that dosage forms such as inappropriate enteric coated, extended-release, and controlled release are used at high rates, such as 9.5%–79.3% [5,17,30,33,34,39]. Abu Hdaib et al. stated that nurses lack knowledge about recognizing dosage forms [30]. It has been emphasized that the insufficient knowledge of nurses about unique drug formulas can threaten the safety of patients because incorrect crushing of such preparations can lead to undesirable patient outcomes and even death [15,40]. Alhashemi et al., in a survey study to determine the knowledge, attitudes, and practices of ICU nurses regarding oral medication administration via EFTs in patients with swallowing difficulties, found that nurses had a deficient awareness of solid dosage form crushing at a rate of 14% [24].

Inadequate awareness of drug dosage forms, especially inappropriate practices, especially in drug administration techniques, may be related to academic education [41]. Moreover, this poor pharmacological knowledge is a regular finding and justification in similar studies [25,40]. On the other hand, the lack of sufficient information in the literature and data from manufacturers has led to using empirical recommendations [25]. Inadequate academic education and limited collaboration among healthcare professionals are key contributors to the observed knowledge gaps. As seen in this study, doctors must support nurses with moderate knowledge of dosage forms. Due to the primary decision-making role of doctors in drug administration via EFT, their inclusion in educational programs and close collaboration between medical teams, including clinical pharmacists and nurses, is essential [23].

### 4.3. Factors affecting knowledge of drug administration through enteral feeding tubes and dosage forms

In this study, it was noteworthy that specialist doctors had significantly higher correct scores. Although doctors did not attend educational programs on drug administration through EFT, their age and professional and ICU experience were higher than nurses. This situation is consistent with the finding that education status did not affect the total number of responses. In the study by Abu Hdaib et al., nurses mostly had 5–10 years of experience, as in this study [30]. In this study, the proportion of nurses who received education on drug administration through EFT was higher than that of nurses in the Abu Hdaib et al. study (12.7%) [30]. The mean age and education status of nurses in the Alahashemi et al. study (86.6% had a bachelor’s degree) were similar to this study. However, their professional and ICU experiences (5 and 2 years) were lower than in this study [24]. However, similarly, the average total knowledge score of nurses was stated as insufficient in other studies, as in this study [24,30]. On the other hand, the factors that most affect nurses’ knowledge about drug administration through EFTs are associated with experience, drug knowledge, hospital policy, consultation with pharmacists, senior or more experienced nurses, and undergraduate education [6,17,25]. Sari et al. reported that almost all nurses (91.3%) acquired knowledge about drug administration through EFTs during their school years, while only 23.2% received in-service education [5]. This underscores the urgent need for educational interventions to improve knowledge levels among healthcare professionals. Studies highlight that the involvement of experts, such as clinical pharmacists and multidisciplinary trainers, can significantly enhance the training of both doctors and nurses, leading to better outcomes in medication administration via EFT [6,17,23,25,41].

Evidence from various studies supports the effectiveness of structured education programs in improving knowledge and practices related to EFT. For example, a quasi-experimental study in four tertiary hospitals in South Korea demonstrated significant improvements in ICU nurses’ knowledge and perception of EFT after a training program, particularly in tube flushing, correct medication administration, and changing feeding sets [42]. Similarly, an intervention study conducted by clinical pharmacists in Iran showed that integrated education programs significantly improved nurses’ knowledge and practice scores compared to a control group [23]. Another prepost virtual training study by Sabaghnejad et al. found that intensive care nurses who received training on EFT had significantly higher knowledge and practice scores than the control group [43]. A prepost intervention study in Jordan further confirmed that structured training significantly enhanced nurses’ knowledge about EFT medication administration [30].

Comprehensive educational programs that address practical challenges and promote adherence to evidence-based guidelines are essential to bridging knowledge gaps [30,34]. A multidisciplinary approach involving collaboration among nurses, doctors, and pharmacists is vital to ensuring patient safety and improving treatment outcomes. Strengthening cooperation among healthcare professionals through targeted education programs can reduce errors, enhance practice quality, and equip teams with the necessary skills for effective drug administration via EFT. Ultimately, the best outcomes will be achieved through the joint efforts of all team members.

### 4.4. Limitations and strengths

Limitations include the exclusion of pharmacists due to their limited presence in ICUs and the study’s restriction to a small number of centers. As far as we searched in the literature, no study has been found that measures the knowledge of ICU doctors about drug administration and dosage form through EFT. In this context, the evaluation of the knowledge level of ICU doctors about drug administrations with EFT is the main strength of this study. Since the study’s findings were conducted in ICUs with similar structures, they can be generalized to other centers. In subsequent studies, a design that includes doctors, nurses, and clinical pharmacists from more centers may be more effective in reflecting the knowledge level of healthcare professionals.

## 5. Conclusion

The knowledge of healthcare professionals in ICUs about drug administrations and dosage form selection via EFT is at a low-medium level. Doctors’ professional and ICU experiences have provided nurses with better knowledge, especially drug dosage forms. We anticipate that sharing doctors’ knowledge with nurses about drug administration via EFT will also contribute to the practice of drug administration via EFT in ICUs.

## Figures and Tables

**Table 1:** Sociodemographic characteristics of healthcare workers in the ıntensive care unit.

Variables	Total (n = 221)	Doctor (n = 112)	Nurse (n = 109)	p
Age, median (IQR)	35 (29–43)	41 (35.25–46)	30 (27–35)	<0.001
Sex, n (%)				0.028
Male	66 (29.9)	41 (36.7)	25 (22.9)	
Female	155 (70.1)	71 (63.3)	84 (87.1)	
Total experience in the profession (years), n (%)				<0.001
1–4 years	45 (20.4)	11 (9.8)	34 (31.2)	
5–10 years	53 (24)	18 (16)	35 (32.2)	
10–15 years	54 (24.4)	30 (26.8)	24 (22)	
>15 years	69 (31.2)	53 (47.4)	16 (14.6)	
Total experience in the intensive care unit (years), median (IQR)	6 (3–10)	7.5 (4–11)	5 (3–9)	0.006
Education level, n (%)				<0.001
Assistant doctor	7 (3.2)	7 (6.2)	0 (0)	
Doctorate	13 (5.9)	13 (11.6)	0 (0)	
Bachelor’s	85 (38.5)	1 (0.8)	84 (77)	
Associate degree	8 (3.6)	0 (0)	8 (7.3)	
Health vocational high school	3 (1.4)	0 (0)	3 (2.7)	
Specialization	84 (38)	84 (38)	0 (0)	
Master’s	21 (9.5)	7 (6.2)	14 (12.8)	
How many hours per week is the training received regarding the preparation and administration of medications? n (%)				<0.001
<1 h	61 (27.6)	19 (16.9)	42 (38.5)	
>10 h	15 (6.8)	6 (5.3)	9 (8.2)	
1–5 h	35 (15.8)	9 (8)	26 (23.8)	
6–10 h	7 (3.2)	3 (2.6)	4 (3.6)	
None	103 (46.6)	75 (66.9)	28 (25.6)	

IQR: Interquartile range

**Table 2:** Evaluation of the correct answer rates for survey questions regarding medication administrations via enteral feeding tubes and appropriate dosage form selection.

Questions and answers	Total (n = 221) (%)	Doctor (n = 112) (%)	Nurse (n = 109) (%)	p
Questions regarding medication applications	**133 (60.1)**	**69 (61.6)**	**64 (58.7)**	**0.471**
1. What is the correct procedure when multiple medications need to be administered to a patient via EFT at the same time? (Medications should be prepared separately and administered separately.)	190 (86)	105 (93.8)	85 (78)	0.001
2. Should liquid dosage forms be diluted before administration via EFT? (Yes.)	107 (48.4)	54 (48.2)	53 (48.6)	1.000
3. Can medications be added directly to the enteral feeding formula? (No, medications should not be added directly to the enteral feeding formula.)	142 (64.3)	76 (67.9)	66 (60.6)	0.285
4. How long should feeding be paused before and after medication administration via EFT? (>30 min)	50 (22.6)	25 (22.3)	25 (22.9)	1.000
5. Which of the following liquid dosage forms should be shaken before use? (Tegretol oral suspension [carbamazepine].)	65 (29.4)	27 (24.1)	38 (34.9)	0.104
6. Is it appropriate to administer liquid medications directly through EFT? (No.)	54 (24.4)	21 (18.8)	33 (30.3)	0.060
7. Should a liquid be administered prior to medication administration to ensure the patency of the EFT? (Yes.)	198 (89.6)	99 (88.4)	99 (90.8)	0.661
8. Should the enteral tube be flushed after medication administration for subsequent interventions? (Yes.)	218 (98.6)	109 (97.3)	109 (100)	0.247
9. Can multiple medications be combined and administered together in the same liquid during simultaneous administration via EFT? (No.)	173 (78.3)	99 (88.4)	74 (67.9)	<0.001
**Questions regarding dosage form selection**	**82 (37.1)**	**49 (43.75)**	**33 (30.2)**	<0.001
10. Which of the following tablets can be crushed? (Film-coated tablets.)	133 (602)	85 (75.9)	48 (44)	<0.001
11. Which of the following capsules/tablets should not be crushed when administered via EFT? (Lansor [Lansoprazole].)	85 (38.5)	37 (33)	48 (4)	0.099
12. Which dosage form of Depakin (Valproic acid) is preferably used for administration via EFT? (Depakin oral solution.)	91 (41.2)	50 (44.5)	41 (37.6)	0.339
13. Are film-coated tablets designed in combination unsuitable for splitting or crushing? (No.)	32 (14.5)	14 (12.5)	18 (16.5)	0.447
14. A drug in the ZOK-release dosage form can be crushed and administered via EFT (such as Beloc ZOK) (No)	76 (34.4)	57 (50.9)	19 (17.4)	<0.001
15. Can a tablet of a CR dosage form of a medication be crushed and administered via EFT (for example, Beloc ZOK)? (No.)	80 (36.2)	49 (43.8)	31 (28.4)	0.025
16. Can all medications in capsule form be administered via EFT by opening/crushing/diluting the capsule? (For example, Lansor capsule.) (No.)	106 (48)	59 (52.7)	47 (43.1)	0.179
17. Are enteric-coated, CR tablets, or capsules prepared by crushing and mixing when administered via EFT? (No.)	90 (40.7)	59 (52.7)	31 (28.4)	<0.001
18. For a patient diagnosed with atrial fibrillation who is prescribed Beloc ZOK (Metoprolol) tablets, which of the following is a more appropriate agent in terms of dosage form and indication for administration via EFT? (Bisoprolol tablet.)	45 (20.4)	30 (26.8)	15 (13.8)	0.019
**Total**	**107 (48.4)**	**58 (51.7)**	**49 (44.9)**	**<0.001**

CR: Controlled release, EFT: Enteral feeding tube, ZOK: Zero order kinetic

**Table 3:** Comparison of the average correct answer rates of healthcare workers for survey questions.

	Total (n = 221) (%)	Doctor (n = 112) (%)	Nurse (n = 109) (%)	p
Total score for medication administrations (mean ± SD)	5.4 ± 1.4	5.4 ± 1.3	5.3 ± 1.4	0.471
Total score for dosage form selection (mean ± SD)	3.3 ± 2.2	3.9 ± 2.1	2.7 ± 1.9	<0.001
Overall total score (mean ± SD)	8.7 ± 2.9	9.4 ± 2.9	8 ± 2.7	<0.001

SD: Standard deviation

## Footnotes

1 Raosoft Sample size calculator [online]. Website https://www.raosoft.com/samplesize.html [accessed 23 October 2024].
